# Characterization of *Ecklonia cava* Alginate Films Containing Cinnamon Essential Oils

**DOI:** 10.3390/ijms19113545

**Published:** 2018-11-10

**Authors:** Su-Kyoung Baek, Sujin Kim, Kyung Bin Song

**Affiliations:** Department of Food Science and Technology, Chungnam National University, Daejeon 34134, Korea; sukyoungb@cnu.ac.kr (S.-K.B.); pppink32@gmail.com (S.K.)

**Keywords:** active packaging materials, alginate films, antimicrobial agents, antioxidant activity, biodegradable films, essential oils

## Abstract

In this study, *Ecklonia cava* alginate (ECA) was used as a base material for biodegradable films. Calcium chloride (CaCl_2_) was used as a cross-linking agent, and various concentrations (0%, 0.4%, 0.7%, and 1.0%) of cinnamon leaf oil (CLO) or cinnamon bark oil (CBO) were incorporated to prepare active films. The ECA film containing 3% CaCl_2_ had a tensile strength (TS) of 17.82 MPa and an elongation at break (E) of 10.36%, which were higher than those of the film without CaCl_2_. As the content of essential oils (EOs) increased, TS decreased and E increased. Addition of CLO or CBO also provided antioxidant and antimicrobial activities to the ECA films. The antioxidant activity of the ECA film with CBO was higher than that of the film containing CLO. In particular, the scavenging activities of the 2,2-diphenyl-1-picrylhydrazyl (DPPH) and 2,2′-azino-bis (3-ethylbenzothiazoline-6-sulphonic acid) (ABTS) radicals in the ECA film containing 1% CBO were 50.45% and 99.37%, respectively. In contrast, the antimicrobial activities against *Escherichia coli* O157:H7, *Salmonella* Typhimurium, *Staphylococcus aureus*, and *Listeria monocytogenes* were superior in the ECA films with CLO. These results suggest that ECA films containing CLO or CBO can be applied as new active packaging materials.

## 1. Introduction

In recent years, environmental pollution caused by plastic waste has worsened, and interest in eco-friendly packaging materials has increased. In particular, biodegradable films have been studied as new packaging materials in the food industry [1,2,3]. As biodegradable film materials, carbohydrates, proteins, and lipids are used individually or in combination.

Seaweeds can grow in abundance, and they are an excellent alternative to replace limited land resources [2]. Among them, *Ecklonia cava* is a brown alga present mainly in the East Asian region [4], and it contains approximately 26% alginate by dry weight [5]. Alginate, which is mainly extracted from brown algae, is non-toxic, relatively inexpensive, and biocompatible, and it can be considered as a base material for biodegradable films [3]. However, *Ecklonia cava* alginate (ECA) has not been studied as a biodegradable film.

Alginate is composed of guluronic acid and mannuronic acid, and their ratio affects alginate film properties. When the proportion of guluronic acid is high, the alginate film is hard and fragile. On the contrary, when the proportion of mannuronic acid is high, elastic and soft gels can be formed [6]. Ionic bonds can be formed by the interactions between divalent cations such as calcium and the carboxyl groups of guluronic acid, and the alginate polymer forms an egg-box structure surrounding the divalent cations [7,8], resulting in films with rigid structures by cross-linking. Therefore, the incorporation of calcium chloride (CaCl_2_) in alginate films can be effective in improving the physical properties of the films.

Active packaging can protect foods during storage and extend their shelf life by the addition of active materials to packaging materials [9]. Essential oils (EOs), classified as “Generally Recognized as Safe” by the Food and Drug Administration, are added as natural active materials to produce active packaging [1]. Among EOs, cinnamon oil is extracted from various parts of cinnamon (*Cinnamomum verum*) such as leaf, bark, flower, and root, but cinnamon leaf oil (CLO) and cinnamon bark oil (CBO) are commonly used as EOs. The major compounds in CLO and CBO are eugenol and trans-cinnamaldehyde, respectively, which contribute to antioxidant and antimicrobial activities [10,11]. CLO and CBO can be added to ECA films as natural active materials to produce active packaging films.

Therefore, this study aimed to prepare alginate films extracted from *Ecklonia cava* with improved physical properties and to characterize the films containing various concentrations of CLO or CBO.

## 2. Results and Discussion

### 2.1. Attenuated Total Reflectance-Fourier Transform Infrared (ATR-FTIR) Analysis of ECA

**Figure 1:**
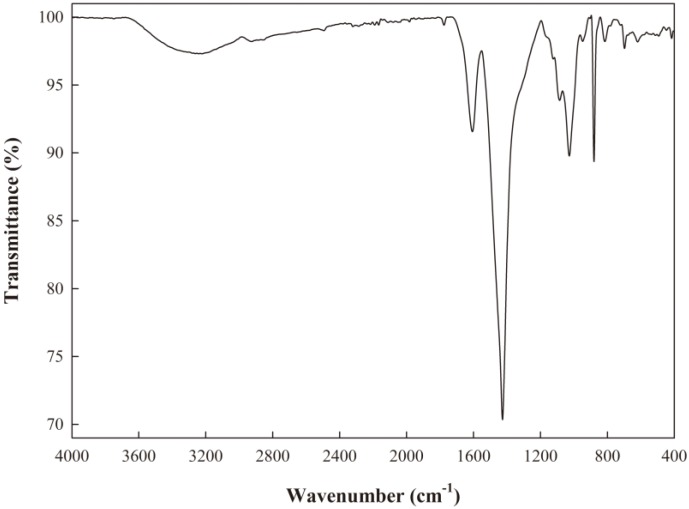
FTIR spectra of *Ecklonia cava* alginate (ECA).

To investigate the molecular characteristics of ECA, ATR-FTIR analysis was performed and the results are presented in Figure 1. A sharp peak at 3600–3500 cm^−1^ indicates the presence of free OH groups [12], but it was not observed in the FTIR spectrum of ECA. The broad band at 3500–3100 cm^−1^ was observed owing to the vibration of the hydroxyl group [12,13]. In the case of alginate, the stretching of protonated carboxyl groups appeared at approximately 1730 cm^−1^. When monovalent ions (sodium) displaced the proton, peaks on the polymeric backbone of alginate appeared at approximately 1600 cm^−1^ and 1400 cm^−1^ by asymmetric and symmetric stretching vibration of the carboxylate group of sodium alginate [8]. In this study, the bands were observed at 1607.01 cm^−1^ and 1426.74 cm^−1^. These results are in accordance with the data found in previous reports on alginate [7,8]. The band at 1027.94 cm^−1^ represented the stretching vibration of C-O, and the band at 880.16 cm^−1^ was related to the C1-H deformation vibration of β-mannuronic acid residues [7]. In general, the content of guluronic acid (G) and mannuronic acid (M) in alginate varies, depending upon the alginate species. Since G and M have characteristic bands at 1025 cm^−1^ and 1100 cm^−1^, respectively, an approximate M/G ratio can be predicted by FTIR analysis [14], and the M/G ratio of ECA was estimated to be lower than 1 in the present study.

### 2.2. Physical Properties of ECA Films with CaCl_2_

The concentration of the cross-linking agent and the type and concentration of the plasticizer have been optimized based on preliminary experiments. In this study, CaCl_2_ was incorporated as a cross-linking agent, and various concentrations of CaCl_2_ (1%, 2%, 3%, 5%, and 7%) were added to the ECA films. The physical properties of the ECA films prepared with varying contents of CaCl_2_ are shown in Table 1. The thickness and tensile strength (TS) of the ECA films with added CaCl_2_ increased compared to those of films without CaCl_2_. In particular, the TS of the ECA film containing 3% CaCl_2_ was significantly improved from 10.49 MPa to 17.82 MPa compared to that of the ECA film without CaCl_2_. The incorporation of CaCl_2_ increased the TS because of the formation of more rigid polymers via interactions between the calcium ions and carboxyl groups of alginate [15]. Zactiti and Kieckbusch [16] reported that increasing calcium concentration resulted in a thicker “egg-box” structure and the formation of a stronger network. Although the addition of CaCl_2_ improved the TS of the ECA film, it did not affect water vapor permeability (WVP). Similar results have been reported in the literature [8,17]. In the present study, the concentration of CaCl_2_ was optimized to 3% based on the mechanical properties of the film.

### 2.3. Physical Properties of ECA Films Containing EOs

EOs were added to ECA films at different concentrations (0.4%, 0.7%, and 1.0%), and Table 2 shows the physical properties of ECA films incorporated with CLO or CBO. The addition of EOs increased the film thickness and elongation at break (E) values, whereas the TS decreased. In particular, the addition of CLO or CBO at 0.7% into the ECA films increased E from 10.36% to 18.25% or 18.65%, respectively, resulting in flexible ECA films. EOs can interfere with the interactions between alginate and calcium ions, thereby improving the flexibility of the films by reducing the intermolecular forces along the polymer chain. Benavides et al. [15] found that, as the content of oregano EOs increased in alginate films, TS decreased and E increased because oregano EOs could act as a plasticizer and increase flexibility and chain mobility. These findings are consistent with those in previous studies on other alginate films containing EOs [18,19].

In general, the addition of hydrophobic EOs causes a decrease in WVP in the films [1]. It has been reported that the incorporation of EOs into various films improved the WVP [20,21,22]. On the contrary, the WVP of ECA films containing CLO or CBO increased as the contents of EOs increased in the present study. This difference can be explained by the formation of pores via changes in the internal structure of the film matrix. Previously, it was reported that the WVP of alginate films incorporated with garlic oil increased as a result of expansion by the intermolecular interactions in the film matrix [19]. Similarly, the WVP of alginate films with other EOs was not improved [18,23]. Atarés et al. [24] also reported that the WVP of the films was not easily improved by the addition of hydrophobic materials, although the incorporation of lipids in the microstructure of the films affected the WVP.

### 2.4. Optical Properties of ECA Films Containing EOs

The optical characteristics of the films are important because they directly affect the appearance and quality of food products. They influence not only customer preference but also the lipid oxidation rate, which can affect the quality of foods [20]. As shown in Table 3, the incorporation of EOs decreased the values of L * and b *, whereas the value of a * increased. The total color difference (ΔE *) in ECA films with EOs increased compared with that in films without EOs. Furthermore, the ECA films gradually became opaque with increasing EO content. The opacity values of ECA films with CLO or CBO at 1.0% were 25.12 or 22.54 A/mm, respectively, whereas ECA films without EOs had an opacity of 9.77 A/mm. Similar to our results, the addition of CBO into chitosan films decreased lightness, whereas redness and opacity increased [25].

### 2.5. Scanning Electron Microscopy (SEM) Analysis

**Figure 2:**
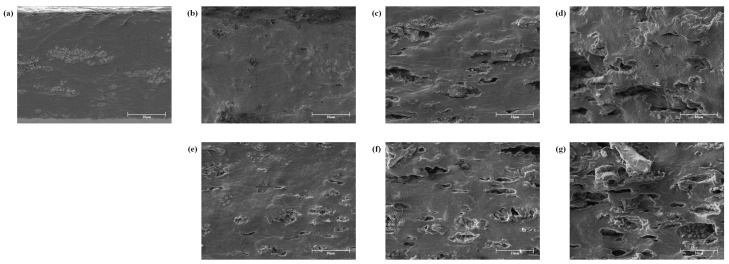
SEM images of the ECA films containing essential oils (EOs). (**a**) Control, (**b**) cinnamon leaf oil (CLO) 0.4%, (**c**) CLO 0.7%, (**d**) CLO 1.0%, (**e**) cinnamon bark oil (CBO) 0.4%, (**f**) CBO 0.7%, and (**g**) CBO 1.0%.

Cross-sectional images showing the internal structure of ECA films incorporated with CLO or CBO are presented in Figure 2. As the content of EOs increased, it was apparent that the microstructure was not compact and pores were formed. The structural change and pore formation in the film increased the WVP. Similarly, when CBO was added to gelatin films, pores were formed by aggregation caused by irregular dispersion of hydrophobic molecules as the concentration of EOs increased [26]. Zhang et al. [23] also reported that alginate films had a relatively homogeneous and smooth surface, but the addition of cinnamon EOs or soybean EOs resulted in irregular surfaces and pores. It was reported that these irregular surfaces and pores were generated by the arrangement of alginate molecules and the agglomeration of oil droplets when the films were formed.

### 2.6. Thermal Analysis

**Figure 3:**
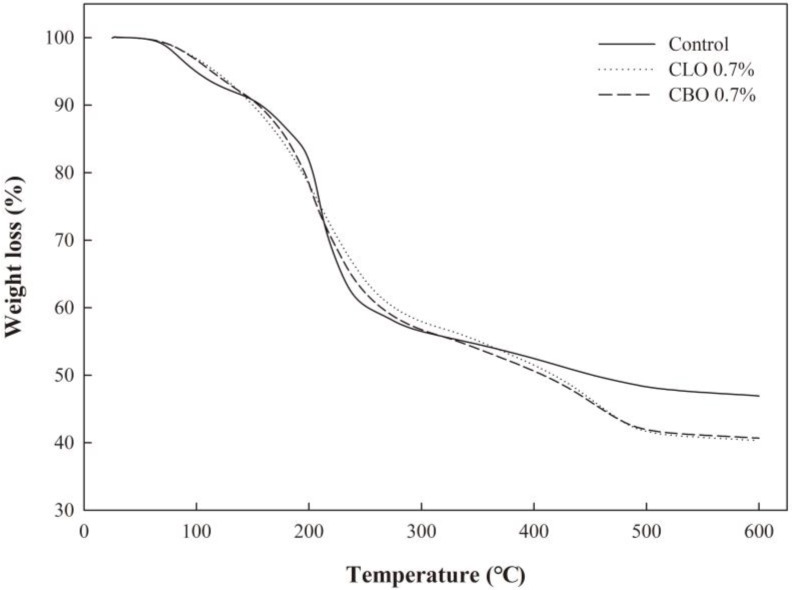
Thermogravimetric analysis (TGA) curves of the ECA films containing cinnamon leaf oil (CLO) and cinnamon bark oil (CBO).

Figure 3 shows the effect of the incorporation of CLO or CBO on the thermal stability of the ECA films using thermogravimetric analysis (TGA). The initial loss of mass appeared to be due to loss of moisture in the ECA film matrix [27]. The main reduction of mass occurred in the range of 200 °C to 300 °C, and the decrease in this region was caused by the volatilization of glycerol used as a plasticizer and the alginate polymer [28]. A loss of mass in ECA films with CLO or CBO was observed at 450 °C, probably due to the presence of highly stable substances in the EOs. At 600 °C, the residue amounts in the control, CLO 0.7%, and CBO 0.7% films were 46.93%, 40.31%, and 40.68%, respectively. The amount of residue was highest in the control, suggesting high thermal stability. The decrease in thermal stability of ECA films incorporated with EOs could be due to the structural change in the ECA film network, causing a decrease in TS by the incorporation of EOs.

**Figure 4:**
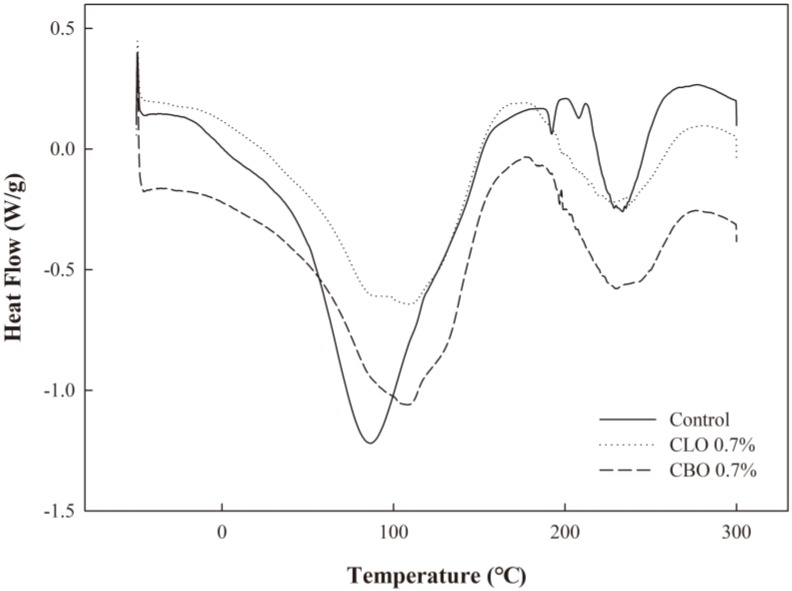
DSC curves of the ECA films containing CLO and CBO.

The results of differential scanning calorimetry (DSC) analysis of the ECA films, the first heating scans, are presented in Figure 4. The ECA film without EOs (control) showed two T_e_ (endothermic peak temperature) at 86.63 (T_e1_) and 233.65 °C (T_e2_). This observation was similar to the results of DSC analysis of alginate having Te at 86 °C and 197 °C [29]. However, it should be noted that the T_e2_ was higher for the ECA film, probably due to the cross-linking effect of CaCl_2_. T_e1_ reflected water loss, and the addition of CLO and CBO to the ECA film increased T_e1_ to 109.81 °C and 108.46 °C, respectively. The ECA films also showed an exothermic peak, and a second endothermic peak T_e2_ appeared at 233.65 °C in the control and at 236.66 °C and 229.14 °C for ECA films with CLO and CBO, respectively. These peaks could be attributed to recrystallization of ECA after heat induction [30]. The values of T_g_ (glass transition temperature) were measured in the second heating scan. In general, the T_g_ of alginate is around 120 °C [31]. In this study, the T_g_ of the control was 126.34 °C and decreased to 103.54 °C and 103.63 °C with the addition of CLO and CBO, respectively. The lower T_g_ was reflected by the decrease in TS due to the addition of EOs in the ECA films, thereby lowering the amount of thermal energy required to transform the ECA films from a glassy to a rubbery state. Tongnuanchan et al. [32] also observed similar results in gelatin films, where EOs interfered with the interactions of polymers and increased the mobility of the polymer by weakening the film structure and consequently lowering T_g_. These results clearly support the TGA results, where the thermal stability of the ECA films with CLO or CBO did not improve compared to the control.

### 2.7. Antioxidant Activity

**Figure 5:**
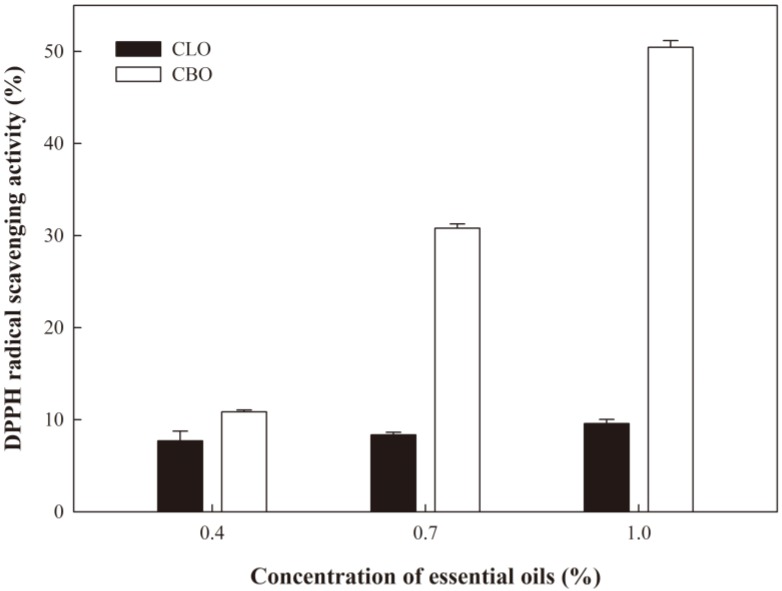
DPPH radical scavenging activity of the ECA films containing EOs.

**Figure 6:**
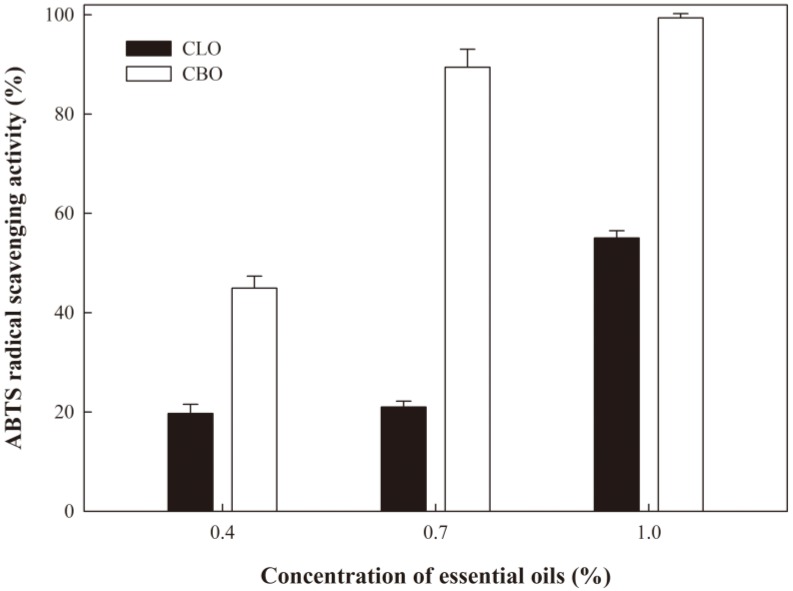
ABTS radical scavenging activity of the ECA films containing EOs.

The antioxidant property of ECA films containing EOs was evaluated (Figure 5 and Figure 6). As the content of CLO or CBO increased, the radical scavenging properties of 2,2-diphenyl-1-picrylhydrazyl (DPPH) and 2,2′-azino-bis (3-ethylbenzothiazoline-6-sulphonic acid) (ABTS) increased. The antioxidant activity of the ECA films with CBO was superior to that of the films with CLO. ECA films containing CLO at various concentrations did not exhibit significant differences in antioxidant activity, except for the film incorporated with 1.0% CLO. In contrast, ECA films with CBO exhibited significant differences in antioxidant activity as the EO content increased. The DPPH radical scavenging activities of ECA films containing CLO or CBO at 1.0% were 9.58% and 50.45%, respectively, and the ABTS radical scavenging activities were 55.05% and 99.37%, respectively. It is known that eugenol has higher radical scavenging activity than trans-cinnamaldehyde [33]. However, the effect of CBO on the radical scavenging activities of DPPH and ABTS was greater than that of CLO in the present study. Singh et al. [34] reported that the DPPH radical scavenging ability of CBO was better than that of CLO, even though eugenol has stronger DPPH radical scavenging ability than trans-cinnamaldehyde. These results suggest that antioxidant activity is affected not only by the major component of the EOs, eugenol or trans-cinnamaldehyde, but also by other constituents in the EOs. Overall, our results suggest that ECA films containing CBO are more suitable as antioxidant films than those containing CLO.

### 2.8. Antimicrobial Activity

Table 4 shows the inhibitory effects of ECA films containing EOs against various bacteria (*Escherichia coli* O157:H7, *Salmonella* Typhimurium, *Staphylococcus aureus*, and *Listeria monocytogenes*). As expected, ECA films without CLO or CBO did not show antimicrobial activity against bacterial strains. In addition, the ECA film, containing 0.4% EO, showed no antimicrobial activity because of the low EO concentration. In contrast, ECA films containing 0.7% and 1.0% EOs exhibited antimicrobial activity, and the inhibition zones against four strains significantly increased as the amount of EOs increased. The antibacterial ability of EOs is mainly due to the presence of functional compounds, such as eugenol and trans-cinnamaldehyde, which disrupt the bacterial cell structure by penetrating cell membranes and inhibiting the production of essential enzymes in bacteria [35]. As shown in Table 3, ECA films containing CLO showed higher antimicrobial activity against four strains, when compared to the films incorporated with CBO. This is probably due to the difference in antimicrobial activity between eugenol, the main component of CLO, and trans-cinnamaldehyde, a major component of CBO [36]. Kang and Song [37] demonstrated the higher antibacterial effect of CLO (higher than that of CBO) by measuring the minimum inhibitory concentration of the EOs.

## 3. Materials and Methods

### 3.1. Materials

*Ecklonia cava* (Jeju-do, Korea) was processed in the laboratory, as described below. Glycerol and CaCl_2_ were purchased from Samchun Pure Chemical Co. (Pyeongtaek-si, Gyeonggi-do, Korea). Span 80 was purchased from Yakuri Pure Chemical Co. (Osaka, Japan). CLO and CBO were obtained from Gooworl Co. (Daegu, Korea).

### 3.2. Alginate Extraction

Alginate extraction from *Ecklonia cava* was performed as reported previously [38,39]. Ground *Ecklonia cava* in 0.025% H_2_SO_4_ solution (1:50, *w*/*v*) was stirred for 3 h. After filtration, the residue was washed with distilled water and filtered again. The residue in 3% Na_2_CO_3_ solution (1:50, *w*/*v*) was heated at 60 °C for 2 h. Distilled water (1:25, *w*/*v*) was added and the mixture was centrifuged at 5000× *g* for 10 min. Methanol (95%, 1:50, *v*/*v*) was added to the supernatant and kept at 4 °C for 1 h. After centrifugation (10,000× *g*, 20 min), the supernatant was removed and the precipitate was lyophilized to obtain ECA.

### 3.3. ATR-FTIR Spectroscopy

FTIR spectrometry (Vertex 80v, Bruker, Billerica, MA, USA) was used to obtain ATR-FTIR spectra of ECA. Analysis was performed with 16 scans for each spectrum in the region between 4000 cm^−1^ and 400 cm^−1^.

### 3.4. Preparation of ECA Films Containing EOs

To prepare the film-forming solution, ECA (1.5%), glycerol (0.3 g/g ECA), and CaCl_2_ (0.03 g/g ECA) were dispersed in distilled water, followed by stirring at 70 °C for 30 min until complete dissolution was achieved. After homogenization (12,000 rpm, 3 min), various concentrations (0.4%, 0.7%, and 1.0% of total volume) of EOs and Span 80 (25% of EOs) were incorporated in the solution. Next, the mixture was homogenized at 12,000 rpm for 7 min, sonicated for 8 min, and degassed for 5 min. The filtered film solution (16 mL) was uniformly spread onto Petri dishes (90 mm diameter) and dried at 25 °C for 18 h.

### 3.5. Physical Properties

A micrometer (No. 7327, Mitutoyo Co., Kawasaki, Japan) was used to measure the thickness of each film. TS and E of the ECA films containing CLO or CBO were evaluated with a Testometric machine (Model 250-2.5 CT, Testometric Co., Lancashire, UK). The films were cut into a rectangular shape (2.54 cm × 10 cm) for the measurement of their physical properties such as TS and E. The initial grip distance in the instrument was 50 mm, and its cross-head speed was set at 50 mm/min. The TS of the films, which is the maximum stress to withstand before breakage and is measured as the highest point of stress–strain curve, was obtained by dividing the maximum force (N), applied when the film was broken, by the cross-sectional area of the film. In addition, the E (%) was calculated by dividing the maximum length (mm) at the moment of film rupture by the initial grip distance (mm), followed by multiplying with 100. The method used for WVP measurement was previously described by Lee and Song [40]. Before the measurement, the water content of the ECA films was controlled by conditioning in a thermos-hygrostat (25 °C, 50% relative humidity) for 24 h.

### 3.6. Optical Properties

To characterize the color of the ECA films containing CLO or CBO, a colorimeter (CR-400M, Minolta, Tokyo, Japan) was used. According to the CIELAB color scale, the L *, a *, and b * values of the prepared ECA films were evaluated on a white standard plate with L *, a *, and b * values of 97.37, −0.14, and 2.00, respectively. The difference in color between the ECA film without EO and those containing EO was indicated as ΔE *. The method described by Yang et al. [41] was used to obtain the opacity of the ECA films with a spectrophotometer (UV-2450, Shimadzu Co., Kyoto, Japan).

### 3.7. SEM Analysis

To investigate the microstructure of the ECA films, SEM analysis was conducted. Before the analysis, the ECA films containing CLO or CBO were fractured in liquid nitrogen and coated with a platinum layer. SEM images of cross sections were acquired using an ion beam scanning electron microscope (LYRA3 WMU, TESCAN, Brno, Czech Republic) under an accelerating voltage of 5 kV. All images were displayed at a magnification of 5000×.

### 3.8. Thermal Property Analysis

To analyze the thermal properties of the ECA films, TGA and DSC were conducted. TGA was carried out with a thermogravimetric analyzer (Mettler Toledo, Columbus, OH, USA). The films (3.8 ± 0.1 mg) were sealed in a sample pan and heated in the temperature range of 25–600 °C at a rate of 10 °C/min under nitrogen flow at 50 mL/min. DSC measurements were carried out using a DSC1 (Mettler Toledo, Schwerzenbach, Switzerland). Each sample was scanned from −50 °C to 300 °C at a rate of 10 °C/min. After the first scan was completed, the second scan was conducted in the same way.

### 3.9. Antioxidant Activity

To examine the antioxidant property of the ECA films, DPPH and ABTS were used. The method described by Shojaee-Aliabadi et al. [42] was used to measure the DPPH radical scavenging activities of the ECA films. The film solution (1%) for this measurement was prepared by diluting the ECA film (0.1 g) in distilled water (10 mL) and shaking in an incubator for 30 min. Next, the solution was mixed with the DPPH solution at a ratio of 1:39 and stored in the dark for 1 h, followed by measuring the absorbance at 517 nm. The ABTS radical scavenging activities of the ECA films were also determined [43]. Before the test, ABTS solution was prepared by blending 7 mM ABTS and 2.45 mM potassium persulfate (2:1). After keeping the solution in the dark for 16 h, ethanol was added to dilute the solution until it had an absorbance value of 0.7 at 734 nm. The ABTS solution was then mixed with the film solution (1%). After being stored in the dark for 10 min, the supernatant of the mixture was separated by centrifugation (3000 rpm, 5 min) and the absorbance was measured at 734 nm.

### 3.10. Antimicrobial Activity

The disc diffusion method was applied to confirm the antibacterial property of the ECA films. *E. coli* O157:H7 (NCTC 12079, ATCC 43889) and *S.* Typhimurium (KCTC 2421, ATCC 14028) were inoculated into tryptic soy broth. *S. aureus* (KCTC 1621, ATCC 10537) and *L. monocytogenes* (KCTC 13064, ATCC 15313) were cultured in brain heart infusion broth. Culture cocktails were prepared by combining each bacterial strain. The bacterial solutions were diluted with peptone water (0.1%) and placed on Mueller Hinton Agar (MHA) medium. The ECA film-forming solutions (80 µL) were impregnated into 8 mm paper discs and dried for 30 min. After drying, the discs were put onto the inoculated MHA medium. Thereafter, it was incubated at 37 °C for 24 h, and the size of the inhibition zone was measured with a digimatic caliper (Model 500-181-20, Mitutoyo Corp., Kawasaki, Japan).

### 3.11. Statistical Analysis

The SAS program (version 9.4, SAS Institute Inc., Cary, NC, USA) was used for statistical analysis of the experimental results. All values are presented as mean ± standard deviations, and *p* < 0.05 indicates statistically significant differences. All experiments were repeated at least 5 times.

## 4. Conclusions

New biodegradable films were prepared using ECA, which has not yet been studied, as a base material in this study. The physical properties of the ECA films were improved with the use of CaCl_2_ as a cross-linking agent, and 3% of CaCl_2_ was found to be optimal. CLO and CBO were incorporated to develop active packaging films and, as a result, have influenced the various properties of the ECA film. As the content of EOs increased, the TS decreased, while E increased. The incorporation of CLO or CBO into the films resulted in antioxidant activities, and the ECA films with CBO showed stronger DPPH and ABTS radical scavenging activities than did the ECA films containing CLO. Antimicrobial activities against various bacteria were confirmed, and higher antimicrobial activity was found in the ECA film containing CLO. Therefore, our studies demonstrate that the ECA films containing CLO or CBO can be applied as new active packaging materials in the food industry.

## Figures and Tables

**Table 1:** Mechanical properties of the *Ecklonia cava* alginate (ECA) films containing different amounts of CaCl_2_.

CaCl_2_ (%)	Thickness (µm)	Tensile Strength (MPa)	Elongation at Break (%)	Water Vapor Permeability (×10^−9^ g /m s Pa)
0	34.60 ± 0.86 ^d^	10.49 ± 1.38 ^d^	9.30 ± 0.89 ^bc^	1.89 ± 0.13 ^a^
1	35.00 ± 0.81 ^cd^	14.78 ± 2.74 ^b^	9.90 ± 0.56 ^ab^	1.86 ± 0.16 ^a^
2	35.96 ± 1.42 ^bc^	12.99 ± 1.65 ^bc^	9.96 ± 0.52 ^ab^	1.85 ± 0.15 ^a^
3	36.68 ± 0.61 ^b^	17.82 ± 1.05 ^a^	10.36 ± 0.27 ^a^	1.78 ± 0.02 ^a^
5	37.12 ± 0.64 ^b^	15.26 ± 1.80 ^b^	9.69 ± 0.60 ^ab^	1.83 ± 0.10 ^a^
7	38.80 ± 1.20 ^a^	10.79 ± 1.55 ^cd^	8.85 ± 0.49 ^c^	1.74 ± 0.07 ^a^

Means ± S.D., *n* = 5, ^a–d^: any means in the same column followed by different letters are significantly (*p* < 0.05) different by Duncan′s multiple range test.

**Table 2:** Physical properties of the ECA films containing essential oils (EOs).

EOs (%)		Thickness (µm)	Tensile Strength (MPa)	Elongation at Break (%)	Water Vapor Permeability (×10^−9^ g /m s Pa)
Control	0	36.68 ± 0.61 ^d^	17.82 ± 1.05 ^a^	10.36 ± 0.27 ^c^	1.78 ± 0.02 ^d^
CLO	0.4	40.12 ± 0.73 ^c^	17.40 ± 0.87 ^ab^	17.94 ± 2.59 ^ab^	2.01 ± 0.05 ^c^
	0.7	44.12 ± 0.67 ^b^	15.10 ± 1.88 ^cd^	18.25 ± 3.32 ^ab^	2.35 ± 0.06 ^b^
	1.0	49.80 ± 0.60 ^a^	14.94 ± 1.22 ^cd^	17.28 ± 1.40 ^ab^	2.56 ± 0.05 ^a^
CBO	0.4	40.40 ± 1.14 ^c^	16.02 ± 0.80 ^bc^	16.92 ± 1.73 ^ab^	1.99 ± 0.03 ^c^
	0.7	44.08 ± 0.61 ^b^	15.45 ± 1.32 ^c^	18.65 ± 2.06 ^a^	2.30 ± 0.07 ^b^
	1.0	49.68 ± 0.46 ^a^	13.58 ± 0.92 ^d^	15.61 ± 1.77 ^b^	2.54 ± 0.09 ^a^

Means ± S.D., *n* = 5, ^a–d^: any means in the same column followed by different letters are significantly (*p* < 0.05) different by Duncan′s multiple range test. CLO: cinnamon leaf oil; CBO: cinnamon bark oil.

**Table 3:** Optical properties of the ECA films containing EOs.

EOs (%)		L *	a *	b *	ΔE *	Opacity (A/mm)
Control	0	72.03 ± 0.27 ^a^	5.16 ± 0.10 ^e^	49.19 ± 0.34 ^ab^	-	9.77 ± 0.31 ^f^
CLO	0.4	68.52 ± 0.40 ^b^	7.71 ± 0.26 ^d^	49.76 ± 0.32 ^a^	4.39 ± 0.48 ^d^	13.43 ± 0.51 ^d^
	0.7	66.40 ± 0.43 ^d^	8.53 ± 0.19 ^b^	45.21 ± 1.08 ^d^	7.75 ± 0.35 ^b^	22.64 ± 0.53 ^b^
	1.0	64.73 ± 1.11 ^e^	8.96 ± 0.66 ^a^	44.55 ± 0.77 ^d^	9.48 ± 1.20 ^a^	25.12 ± 0.37 ^a^
CBO	0.4	68.48 ± 0.33 ^b^	7.52 ± 0.19 ^d^	49.00 ± 0.97 ^ab^	4.37 ± 0.36 ^d^	12.12 ± 0.19 ^e^
	0.7	67.12 ± 1.10 ^c^	8.17 ± 0.24 ^c^	48.52 ± 0.81 ^b^	5.86 ± 1.10 ^c^	20.85 ± 0.34 ^c^
	1.0	64.01 ± 0.67 ^f^	8.59 ± 0.42 ^b^	46.87 ± 1.29 ^c^	9.11 ± 0.79 ^a^	22.54 ± 0.54 ^b^

Means ± S.D., *n* = 10, ^a–f^: any means in the same column followed by different letters are significantly (*p* < 0.05) different by Duncan′s multiple range test.

**Table 4:** Antimicrobial activity of the ECA films containing EOs.

EOs (%)		Inhibition Zone (mm)			
		*E. coli* O157:H7	*S.* Typhimurium	*S. aureus*	*L. monocytogenes*
Control	0	ND *	ND	ND	ND
CLO	0.4	ND	ND	ND	ND
	0.7	12.04 ± 0.10 ^c^	11.49 ± 0.14 ^c^	10.77 ± 0.07 ^b^	13.17 ± 0.49 ^b^
	1.0	14.50 ± 0.51 ^a^	15.37 ± 0.16 ^a^	14.28 ± 0.25 ^a^	15.02 ± 0.17 ^a^
CBO	0.4	ND	ND	ND	ND
	0.7	10.88 ± 0.08 ^d^	10.77 ± 0.09 ^d^	10.35 ± 0.52 ^b^	12.03 ± 0.66 ^c^
	1.0	13.48 ± 0.17 ^b^	12.76 ± 0.22 ^b^	13.93 ± 0.36 ^a^	14.48 ± 0.13 ^a^

Means ± S.D., *n* = 5, ^a–d^: any means in the same column followed by different letters are significantly (*p* < 0.05) different by Duncan′s multiple range test, * ND: not detected.

## References

[1] Atarés L., Chiralt A. (2016). Essential oils as additives in biodegradable films and coatings for active food packaging. Trends Food Sci. Technol..

[2] Khalil H.A., Tye Y.Y., Saurabh C.K., Leh C.P., Lai T.K., Chong E.W.N., Syakir M.I. (2017). Biodegradable polymer films from seaweed polysaccharides: A review on cellulose as a reinforcement material. Express Polym. Lett..

[3] Tavassoli-Kafrani E., Shekarchizadeh H., Masoudpour-Behabadi M. (2016). Development of edible films and coatings from alginates and carrageenans. Carbohydr. Polym..

[4] Wijesinghe W.A.J.P., Jeon Y.J. (2012). Exploiting biological activities of brown seaweed *Ecklonia cava* for potential industrial applications: A review. Int. J. Food Sci. Nutr..

[5] Park Y.H. (1969). Seasonal variation in the chemical composition of brown algae with special reference to alginic acid. Korean J. Fish. Aquat. Sci..

[6] Jost V., Reinelt M. (2018). Effect of Ca^2+^ induced crosslinking on the mechanical and barrier properties of cast alginate films. J. Appl. Polym. Sci..

[7] Fawzy M.A., Gomaa M., Hifney A.F., Abdel-Gawad K.M. (2017). Optimization of alginate alkaline extraction technology from *Sargassum latifolium* and its potential antioxidant and emulsifying properties. Carbohydr. Polym..

[8] Sellimi S., Younes I., Ayed H.B., Maalej H., Montero V., Rinaudo M., Nasri M. (2015). Structural, physicochemical and antioxidant properties of sodium alginate isolated from a Tunisian brown seaweed. Int. J. Biol. Macromol..

[9] Kuorwel K.K., Cran M.J., Orbell J.D., Buddhadasa S., Bigger S.W. (2015). Review of mechanical properties, migration, and potential applications in active food packaging systems containing nanoclays and nanosilver. Compr. Rev. Food Sci. F..

[10] Ranasinghe P., Pigera S., Premakumara G.S., Galappaththy P., Constantine G.R., Katulanda P. (2013). Medicinal properties of ‘true’cinnamon (*Cinnamomum zeylanicum*): A systematic review. BMC Complement Altern. Med..

[11] Sangal A. (2011). Role of cinnamon as beneficial antidiabetic food adjunct: A review. Adv. Appl. Sci. Res..

[12] Mahmood A., Bano S., Kim S.G., Lee K.H. (2012). Water–methanol separation characteristics of annealed SA/PVA complex membranes. J. Memb. Sci..

[13] Falkeborg M., Paitaid P., Shu A.N., Pérez B., Guo Z. (2015). Dodecenyl succinylated alginate as a novel material for encapsulation and hyperactivation of lipases. Carbohydr. Polym..

[14] Pereira L., Sousa A., Coelho H., Amado A.M., Ribeiro-Claro P.J. (2003). Use of FTIR, FT-Raman and 13 C-NMR spectroscopy for identification of some seaweed phycocolloids. Biomol. Eng..

[15] Benavides S., Villalobos-Carvajal R., Reyes J.E. (2012). Physical, mechanical and antibacterial properties of alginate film: Effect of the crosslinking degree and oregano essential oil concentration. J. Food Eng..

[16] Zactiti E.M., Kieckbusch T.G. (2006). Potassium sorbate permeability in biodegradable alginate films: Effect of the antimicrobial agent concentration and crosslinking degree. J. Food Eng..

[17] Zactiti E.M., Kieckbusch T.G. (2009). Release of potassium sorbate from active films of sodium alginate crosslinked with calcium chloride. Packag. Technol. Sci..

[18] Han Y., Yu M., Wang L. (2018). Physical and antimicrobial properties of sodium alginate/carboxymethyl cellulose films incorporated with cinnamon essential oil. Food Packag. Shelf Life.

[19] Pranoto Y., Salokhe V.M., Rakshit S.K. (2005). Physical and antibacterial properties of alginate-based edible film incorporated with garlic oil. Food Res. Int..

[20] Abdollahi M., Rezaei M., Farzi G. (2012). Improvement of active chitosan film properties with rosemary essential oil for food packaging. Int. J. Food Sci. Technol..

[21] Shojaee-Aliabadi S., Hosseini H., Mohammadifar M.A., Mohammadi A., Ghasemlou M., Hosseini S.M., Khaksar R. (2014). Characterization of κ-carrageenan films incorporated plant essential oils with improved antimicrobial activity. Carbohydr. Polym..

[22] Tongnuanchan P., Benjakul S., Prodpran T. (2012). Properties and antioxidant activity of fish skin gelatin film incorporated with citrus essential oils. Food Chem..

[23] Zhang Y., Ma Q., Critzer F., Davidson P.M., Zhong Q. (2015). Physical and antibacterial properties of alginate films containing cinnamon bark oil and soybean oil. LWT Food Sci. Technol..

[24] Atarés L., De-Jesús C., Talens P., Chiralt A. (2010). Characterization of SPI-based edible films incorporated with cinnamon or ginger essential oils. J. Food Eng..

[25] Peng Y., Li Y. (2014). Combined effects of two kinds of essential oils on physical, mechanical and structural properties of chitosan films. Food Hydrocoll..

[26] Wu J., Sun X., Guo X., Ge S., Zhang Q. (2017). Physicochemical properties, antimicrobial activity and oil release of fish gelatin films incorporated with cinnamon essential oil. Aquac. Fish..

[27] Yadav M., Rhee K.Y., Park S.J. (2014). Synthesis and characterization of graphene oxide/carboxymethylcellulose/alginate composite blend films. Carbohydr. Polym..

[28] Shankar S., Wang L.F., Rhim J.W. (2016). Preparations and characterization of alginate/silver composite films: Effect of types of silver particles. Carbohydr. Polym..

[29] Soares J.P., Santos J.E., Chierice G.O., Cavalheiro E.T.G. (2004). Thermal behavior of alginic acid and its sodium salt. Eclética Química.

[30] Pongjanyakul T., Priprem A., Puttipipatkhachorn S. (2005). Investigation of novel alginate−magnesium aluminum silicate microcomposite films for modified-release tablets. J. Control Release..

[31] Naidu B.V.K., Sairam M., Raju K.V., Aminabhavi T.M. (2005). Thermal, viscoelastic, solution and membrane properties of sodium alginate/hydroxyethylcellulose blends. Carbohydr. Polym..

[32] Tongnuanchan P., Benjakul S., Prodpran T. (2014). Structural, morphological and thermal behavior characterizations of fish gelatin film incorporated with basil and citronella essential oils as affected by surfactants. Food Hydrocoll..

[33] Sharma U.K., Sharma A.K., Pandey A.K. (2016). Medicinal attributes of major phenylpropanoids present in cinnamon. BMC Complement Altern. Med..

[34] Singh G., Maurya S., Catalan C.A. (2007). A comparison of chemical, antioxidant and antimicrobial studies of cinnamon leaf and bark volatile oils, oleoresins and their constituents. Food Chem. Toxicol..

[35] Sanla-Ead N., Jangchud A., Chonhenchob V., Suppakul P. (2012). Antimicrobial Activity of cinnamaldehyde and eugenol and their activity after incorporation into cellulose-based packaging films. Packag. Technol. Sci..

[36] Nazzaro F., Fratianni F., De Martino L., Coppola R., De Feo V. (2013). Effect of essential oils on pathogenic bacteria. Pharmaceuticals.

[37] Kang J.H., Song K.B. (2018). Inhibitory effect of plant essential oil nanoemulsions against *Listeria monocytogenes*, *Escherichia coli* O157: H7, and *Salmonella* Typhimurium on red mustard leaves. Innov. Food Sci. Emerg. Technol..

[38] Cho M., Yoon S.J., Kim Y.B. (2013). The nutritional composition and antioxidant activity from *Undariopsis peterseniana*. Ocean Polar Res..

[39] You B.J., Jeong I.H., Lee K.H. (1997). Effect extraction conditions on bile acids binding capacity in vitro of alginate extracted from sea tangle (*Laminaria* spp.). Korean J. Fish. Aquat. Sci..

[40] Lee K.Y., Song K.B. (2017). Preparation and characterization of an olive flounder (*Paralichthys olivaceus*) skin gelatin and polylactic acid bilayer film. J. Food Sci..

[41] Yang S.Y., Lee K.Y., Beak S.E., Kim H., Song K.B. (2017). Antimicrobial activity of gelatin films based on duck feet containing cinnamon leaf oil and their applications in packaging of cherry tomatoes. Food Sci. Biotechnol..

[42] Shojaee-Aliabadi S., Hosseini H., Mohammadifar M.A., Mohammadi A., Ghasemlou M., Ojagh S.M., Khaksar R. (2013). Characterization of antioxidant-antimicrobial κ-carrageenan films containing *Satureja hortensis* essential oil. Int. J. Biol. Macromol..

[43] Bitencourt C.M., Fávaro-Trindade C.S., Sobral P.J.A., Carvalho R.A. (2014). Gelatin-based films additivated with curcuma ethanol extract: Antioxidant activity and physical properties of films. Food Hydrocoll..

